# Multidisciplinary cancer disease classification using adaptive FL in healthcare industry 5.0

**DOI:** 10.1038/s41598-024-68919-1

**Published:** 2024-08-12

**Authors:** Tahir Abbas, Areej Fatima, Tariq Shahzad, Meshal Alharbi, Muhammad Adnan Khan, Arfan Ahmed

**Affiliations:** 1 School of Computer Science, National College of Business Administration and Economics, Lahore, 54000 Pakistan; 2 Department of Computer Science, Lahore Garrison University, Lahore, 54000 Pakistan; 3 Department of Computer Sciences, COMSATS University Islamabad, Sahiwal Campus, Sahiwal, 57000 Pakistan; 4 Department of Computer Science, College of Computer Engineering and Sciences, Prince Sattam Bin Abdulaziz University, 11, 11942 Alkharj, Saudi Arabia; 5 Department of Software, Faculty of Artificial Intelligence and Software, Gachon University, Seongnam-si, 13557 South Korea; 6 AI Center for Precision Health, Weill Cornell Medicine-Qatar, P.O. Box 24144, Doha, Qatar

**Keywords:** Federated learning, Adaptive federated learning, Histopathology, Image processing, Brain cancer, Kidney cancer, Breast cancer, Cancer, Kidney diseases, Computational science, Computer science, Information technology

## Abstract

Emerging Industry 5.0 designs promote artificial intelligence services and data-driven applications across multiple places with varying ownership that need special data protection and privacy considerations to prevent the disclosure of private information to outsiders. Due to this, federated learning offers a method for improving machine-learning models without accessing the train data at a single manufacturing facility. We provide a self-adaptive framework for federated machine learning of healthcare intelligent systems in this research. Our method takes into account the participating parties at various levels of healthcare ecosystem abstraction. Each hospital trains its local model internally in a self-adaptive style and transmits it to the centralized server for universal model optimization and communication cycle reduction. To represent a multi-task optimization issue, we split the dataset into as many subsets as devices. Each device selects the most advantageous subset for every local iteration of the model. On a training dataset, our initial study demonstrates the algorithm's ability to converge various hospital and device counts. By merging a federated machine-learning approach with advanced deep machine-learning models, we can simply and accurately predict multidisciplinary cancer diseases in the human body. Furthermore, in the smart healthcare industry 5.0, the results of federated machine learning approaches are used to validate multidisciplinary cancer disease prediction. The proposed adaptive federated machine learning methodology achieved 90.0%, while the conventional federated learning approach achieved 87.30%, both of which were higher than the previous state-of-the-art methodologies for cancer disease prediction in the smart healthcare industry 5.0.

## Introduction

Between different kinds of diseases, cancer is known as the second deadliest disease in the world. More than 9.95 million deaths^1^ occurred among 19.2 million new cancer cases that were reported in 2020. The human body is made up of thousands of live cells, but because the body requires additional cells, the cells divide and multiply. The cells in the body are naturally destroyed and replaced by the new cells this process occurs at a specific age. If these dead cells are not replaced by the body, then they start to multiply and increase in number and the result of this increase in the dead cell causes a tumor. Death from colon and pulmonary cancer occurs in both genders. In 2020, it is anticipated that these lung and colon cancer types will result in 2.75 million deaths and 4.14 million new illnesses worldwide^2^. To overcome these kinds of cancer diseases a smart healthcare system is required that helps in early-stage detection by using smart devices^3^. The Internet of Medical Things (IoMT) is the new emerging technique through which smartphones, laptops, and other hand-held devices can communicate through networks to collect information about patients and detect diseases in their early stages^4^. These gadgets have crucial characteristics like habit tracking, safety tests, and other aspects that encourage users and system developers to carry out in-depth studies^5^. The important thing in this system is the privacy, security, and confidentiality that mainly needs to be resolved. To address these problems and to provide a solution to these problems federated learning (FL) is the answer that can provide all the features that are required by the system in terms of privacy, security, and confidentiality.

Federated learning (FL) is the way to go when it comes to addressing important issues like information-privileged access, data protection, and privacy protection.

Federated learning systems have actively been researched in a variety of environments. Some early publications^6–8^, depict the architecture as a universal common model trained across multiple intelligent devices under the supervision of a central server. The federated model has several notable advantages, including the circulation of processing control to edge nodes and the safeguarding of data privacy. Newly, diverse setups have been anticipated and studied from several angles^9–11^. The resource availability fluctuates at runtime during federated aggregation, and^10^ described it as limiting the optimization issue. We assume, in our proposed framework, that the hospital image data can be retrieved from any device in the smart hospital, which results in an easing of the virtuously distributed architecture. Our research emphasizes the healthcare settings of federated aggregation and improved precision attained through the architecture’s self-adaptive behavior. The development of an Internet of Medical Things (IoMT) application for early detection of sepsis using Raspberry Pi and Jetson Nanodevices^12^ is presented in this study. The application uses a federated multi-modal deep learning approach to analyze Electronic Health Records (EHRs) and detect sepsis in real-time. The study demonstrates the potential of IoMT devices in improving sepsis diagnosis and ultimately reducing the sepsis-related mortality rate. The paper suggests a federated learning ecosystem powered by blockchain to secure consumer IoT feature analysis^13^. The ecosystem integrates blockchain technology to improve the privacy and security of consumer IoT devices by enabling decentralized and secure data sharing between devices. The federated learning approach allows for the training of machine learning models without having to centralize data, thereby improving the privacy of the data. The authors describe the implementation of the ecosystem and demonstrate its effectiveness in securing IoT features analysis through a case study. The study highlights the potential of the proposed approach in addressing privacy and security concerns in IoT devices.

CNNs are a type of neural network commonly used for image recognition and classification tasks. The training process of a CNN involves the following steps: (1) Data Preparation: The training data is usually split into batches, and each batch is passed through the network during training, (2) Forward Propagation: In this step, the input data is fed into the network, and the network performs a series of operations on it to produce an output. These operations typically include convolutions, pooling, and activation functions, (3) Loss Calculation: The network’s performance is gauged by calculating the loss, which is the result of comparing the output to the ground truth labels (4) Backward Propagation: The network uses the loss to update its parameters through backpropagation. This involves calculating the gradients of the loss concerning each parameter and adjusting the parameters accordingly, (5) Optimization: Ultimately, a stochastic gradient descent (SGD) or Adam optimization approach is used to update the network’s parameters.

Federated learning is a distributed machine learning technique that works without requiring data sharing with a central server, allowing several devices to work together to train a model. The main advantage of using CNNs in federated learning is their ability to learn complex representations from raw input data, making them well-suited for tasks such as image recognition. In the context of federated learning, CNNs can be trained using a similar process. Local training and global aggregation are the two main stages of the training process in federated learning, where the data is dispersed over several hospitals instead of devices. Each device uses the above-described standard training procedure to train the CNN on its local data during the local training phase. However, since each device has a different dataset, the network may converge to different solutions on each device. In the global aggregation phase, the network parameters from each device are aggregated to create a global model. This can be done using various techniques such as simple averaging, weighted averaging, or more sophisticated techniques such as Federated Averaging.

Adaptive Federated Learning (AFL) is an extension of FL that enables each device to adapt the global model according to its local data distribution and model capacity. Here are some advantages of AFL over conventional FL: (I) AFL can improve the model accuracy by allowing each device to customize the global model based on its local data distribution. This way, the global model can better fit the data from all devices, resulting in higher accuracy, (II) in conventional FL, the global model is updated only after collecting gradients from all devices, which can be time-consuming. AFL, on the other hand, allows each device to update the global model locally, which can speed up the convergence process, (III) in conventional FL, all devices need to communicate their gradients with a central server, which can lead to high communication overhead. AFL reduces this overhead by allowing devices to update the global model locally, (IV) AFL can increase privacy by allowing each device to keep its data local and update the global model without sharing its data. This way, sensitive data is not shared with other devices or a central server, (V) in conventional FL, all devices are assumed to have the same data distribution and model capacity, which is not always the case in real-world scenarios. AFL can handle device heterogeneity by allowing each device to adapt to the global model based on its local data distribution and model capacity. It is important to note that AFL requires careful design and tuning of several parameters, such as the learning rate, the selection of devices for each iteration, and the local update algorithm. Additionally, AFL requires robust mechanisms for privacy protection, as the local models can contain sensitive information from each device's data.

Overall, AFL can provide several advantages over conventional FL, including improved model accuracy, faster convergence, lower communication overhead, increased privacy, and robustness to device heterogeneity.

The main contribution of this research is as follows:

- Ensuring the security and privacy of “patients” data and developing a safe, automated e-healthcare system are the primary objectives of this research.
- For accurate trans-disciplinary disease identification and treatment, the suggested adaptive federated machine learning approach offers a better option.
- The proposed adaptive FL model is simulated on fused datasets of multidisciplinary cancer disease.

The breakdown of the paper’s structure is as follows: The current advancement in cancer disease monitoring and detection is highlighted in section “Related work” of the literature. Section “Proposed intelligent system for multidisciplinary cancer disease prediction” covers the study methodologies, data set selection, feature extraction, feature selection, and suggested Adaptive Federated Learning approach. Section “Results and discussion” presents the dataset selection, preprocessing, results & discussion. Section Conclusions discusses the conclusion and future work. Citations are provided in section “Contribution and future work” of the paper.

## Related work

According to a recent study, cloud-based medical records have several drawbacks, most of which are related to the way that data about healthcare or medicine is gathered and evaluated from many databases that are accessible from any location. Moreover, there is no infrastructure in place that securely stores and makes all medical and healthcare-related data, such as test results, imaging, or a patient’s prescriptions, accessible from any location. Many medical-related departments now manage data using computer systems and software instead of a manual approach, which cuts down on the amount of human labor and the time and effort required to manually gather data. To access data online from the comfort of their homes, customers still need to travel to the place, which takes time. The responsibilities and activities connected with smart homes have expanded due to recent advancements in information and communication technology (ICT) and the Internet of Things^14^.

A smart healthcare system is a house that continuously gathers and shares data. Smart technology can provide data and automated services from several medical devices, including blood pressure, glucose, and ECG monitors, as well as smartwatches. The community's interactive health computer system automatically incorporates systems that utilize this new technology^15^. To maximize the benefits of health product design, customers may be able to choose how they utilize different medical devices to track and manage their health, depending on their settings and the setup of the smart healthcare network. Thanks to a recent development, it is now possible to run appliances across gates, both within and outside the building^16^. It is expected that when 5G, or fifth-generation mobile networking technology, becomes more prevalent, several industries will merge, hardware will advance, and smart healthcare systems will become more streamlined and organized. Data has become the primary source of knowledge in recent years, and astute solutions to real-world issues in fields like wireless networking, bioinformatics, agribusiness, and finance^17^ have opened up new avenues. Users may complete tasks more quickly thanks to the clear and simple information provided by these data-driven solutions. Using the Precision-weighted FL^18^ method, images in the MNIST dataset were identified. The author discusses attack detection^19,20^ in automated e-healthcare monitoring systems for physical and medical systems that use FL. All communication was done using network devices connected to link nodes to enhance patient care. Owing to the variety of devices linked to automated systems, there was a chance that these devices may be used in a cyber-attack^19^. Hospitalization prediction was created by FL of predictive models in electronic health records (EHR) utilizing a decentralized optimization methodology^21,22^. The author of this work contributes to slowing down the rate of convergence and communication costs. For IoT applications, a bespoke FL^23^ built on the cloud-edge architecture was available. The cloud edge architecture for the Personalized FL framework was first presented in this publication.

This study presents the creation of an anomaly prediction service for software-defined networks (SDNs)^24^, based on machine learning. Networks with separate control and data planes, or SDNs, enable centralized network management and programmability. SDN anomalies can be caused by several things, including hardware malfunctions, malicious assaults, and incorrect configurations. The suggested system analyses network traffic data and looks for anomalies using machine learning techniques. Because it has been educated on typical traffic patterns, the system can recognize departures from them instantly. The system can take the necessary steps, like stopping traffic or notifying the network administrator, once an abnormality is discovered. The article presents experimental results that demonstrate the effectiveness of the proposed system in detecting anomalies with high accuracy and low false positive rates. The authors suggest that the system can be integrated with existing SDN controllers and used in real-world networks to improve network security and reliability.

A thorough investigation of the diagnosis of brain tumors has been carried out using deep and federated learning methodologies^25,26^. The methodology, datasets, and classifiers used in brain tumor research are all thoroughly analyzed in this work. This piece displays in-depth brain tumor analysis based on learning^27^. This research study's key contribution is a thorough examination of brain tumors, their evolution, and the challenges that will be faced in the future when diagnosing and foretelling brain cancers in people. To diagnose brain tumors, this work examines feature selection and segmentation^28^ in MRI images.

More and more doctors are using vigorous contrast-boosted magnetic resonance imaging to discover lesions in the breast when diagnosing breast cancer. However, because the 4D spatial–temporal DCE-MRI^29–32^ data are so large and complex, the diagnosis procedure is lengthy and frequently incorrect. There are situations when BPE makes typical fibroglandular tissue look better, which can harm current algorithms. A 3D Clifford analytic signal (CAS) approach is proposed for the separation of breast lesions from DCE-MRI data. Every single 2D DCE-MRI slice from a specific transverse plane, taken at various scanning times, is employed in the creation of the temporal 2D picture using the CAS approach. To create a 3D Clifford temporal image^30^, temporal images are piled on top of each other (CTI). With the proposed CTI, it is possible to distinguish between areas of lesions both visually and numerically. A fully convolutional network (FCN) model is used as one of the inputs to differentiate breast lesions. TCIA QIN breast DCE-MRI and a private home breast DCE-MRI dataset (TBD) demonstrate that the proposed method outperforms current methods in terms of both quality and quantity.

Clinical research and healthcare services increasingly make use of medical big data for secondary purposes. It is typical for cancer to return after treatment (recovery period). Cancer sufferers feel that studying how often cancer returns and what causes it can lead to beneficial clinical interventions. A total of 50,000 cases of seven different cancers^26,33–36^, including liver, lung, kidney, breast, uterine, stomach, and intestine cancers, were studied for this study. A regression vector machine (TSVR)^37^ based on DNN weighting for twin support is presented herein. With the use of a DNN-weighted approach that incorporates a local information mining function, the eplion-TSVR model’s solution is discovered. For determining the appropriate DNN settings, it is recommended that the Cuckoo method be employed. The updated TSVR algorithm is used in this article to create a model for predicting when cancer may return. In comparison to the convolutional neural network and e-TSVR models, the model’s predictions for different forms of cancer can reach more than 91% accuracy.

## Proposed intelligent system for multidisciplinary cancer disease prediction

**Figure 1:**
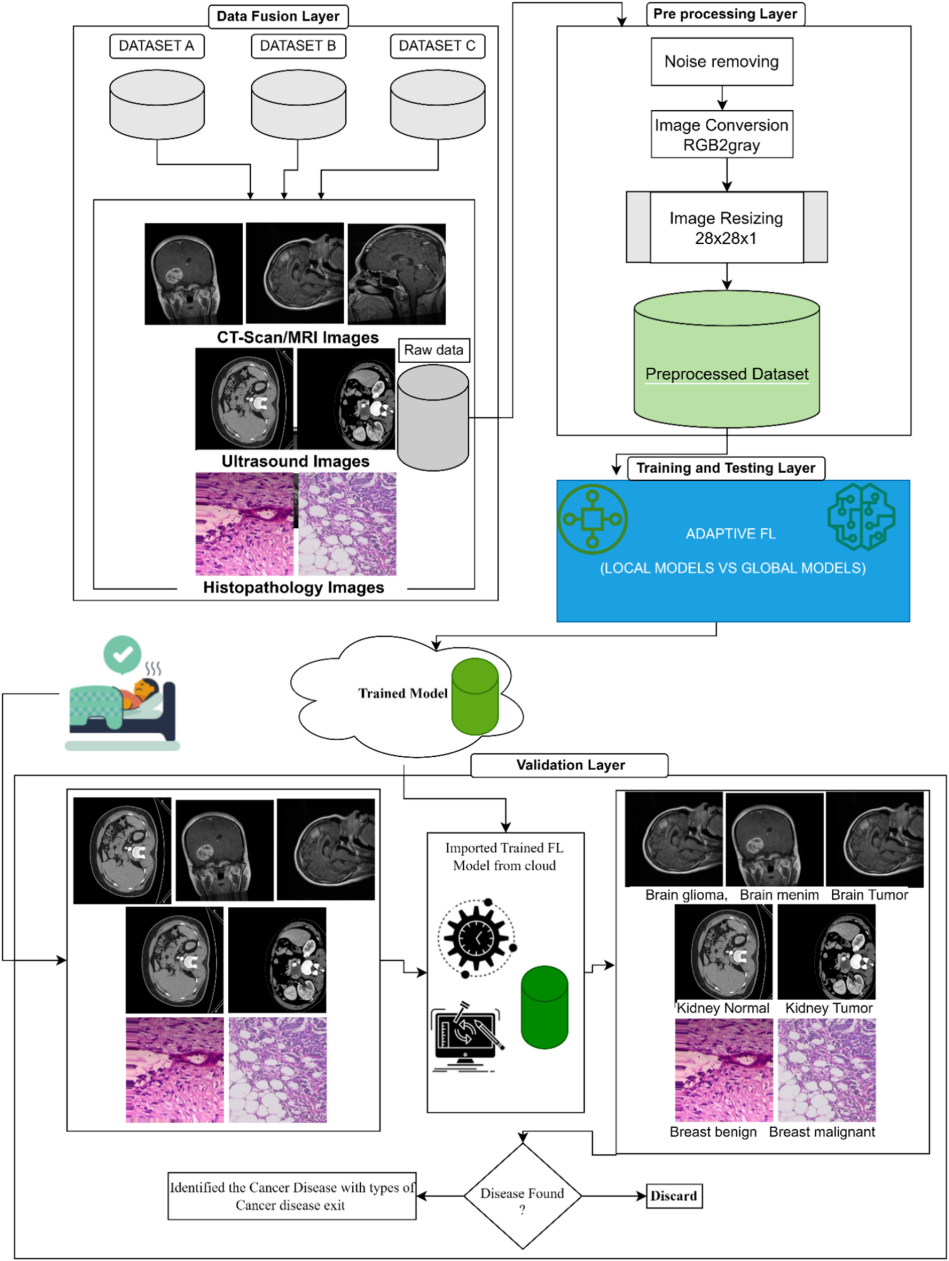
Proposed adaptive federated machine learning Model.

In this Proposed Intelligent System for Multidisciplinary Cancer Disease Prediction, It presents how adaptive federated learning is used in the suggested intelligent system for transdisciplinary cancer illness prediction in the healthcare industry 5.0. In Fig. 1, the suggested model is shown. The stages of the suggested Intelligent System with an adaptive federated machine learning model for smart healthcare system-based multidisciplinary cancer patient prediction are as follows:

The proposed model is divided into four (04) phases; (1) Data fusion Layer, (2) Preprocessing Layer, (3) Training and Testing Layer, and (4) Validation Layer. In the first phase of the proposed model, we have considered the three datasets of cancer diseases named (i) brain cancer, (ii) kidney cancer, and (iii) breast cancer. The brain cancer dataset is further divided into three subtypes of cancer disease. The kidney cancer dataset has two subtypes and the breast cancer dataset also has two subtypes. All the datasets comprised 35,000 images and 5000 images in each class. The detail is shown in Table 1.

**Table 1:** Dataset description.

Sr. #	Dataset	Subtypes	Total images
1	Brain dataset (^38^)	Brain glioma	5000
		Brain menim	5000
		Brain tumor	5000
2	Kidney dataset (^38^)	Kidney normal	5000
		Kidney tumor	5000
3	Breast dataset (^38^)	Breast Benign	5000
		Breast malignant	5000

In the preprocessing layer, the fused dataset is being processed for further use in disease classification in the proposed intelligent system. A collection of procedures and techniques used on digital images before additional processing or analysis is referred to as image preprocessing. Enhancing the image’s quality is the main objective of image preprocessing, as it facilitates the extraction of valuable information by algorithms. Various methods can be applied singly or in combination, based on the particular demands of the image processing assignment. The quality and usefulness of the image for additional analysis can be significantly increased by using the necessary techniques, producing more accurate and trustworthy results.

In 2nd step, we converted all the images from RGB to grayscale to minimize the computation cost. In 3rd step, we resized the images and made the complete fused dataset smooth for further training and training of this dataset. Due to the image’s dataset, the computation cost increases with the increase of images. In our experimentation, we have considered the image size 28*28*1 for fast feature extraction and internal calculations. After completing these three (03) steps in preprocessing layers, the fused dataset is ready for experimentation. In the training and testing layer, a federated learning methodology is adopted for training and testing the dataset of multidisciplinary cancer disease classification. In our proposed adaptive federated learning, the datasets are trained as per the federated learning methodology as discussed in section “Introduction”. All the local models’ weights are exchanged with a global model for synchronization and making the accuracy level equal to each local model as per the global model. In this proposed adaptive federated learning approach, we considered three hospitals having two (02) devices installed, in each hospital, for collecting the data on each cancer disease. The dataset of each smart hospital is distributed for the training and testing model.

The detail of working and mathematical demonstration^39^ is as under and used notation is shown in Table 2.

**Table 2:** Main notation.

${\varvec{H}}$	Number of hospitals
$H_{D} \left( i \right)$	Number of smart devices for hospitals i
${\mathcal{H}}$	Set of all hospitals $\left| {\mathcal{H}} \right|$= H
$T$	Number of global rounds
$L$	Number of local rounds
$G_{i}$	Loss function for the ith hospital
$E_{i}$	Data set for the ith hospital
$l_{jk}$	Loss of the model j for the kth subset
$l_{j}$	Mean loss of the model j on Data Set Ei
${\mathcal{H}}_{{\text{i}}}$	Set of devices for hospital i

Consider the set ${\mathcal{H}} = \left\{ {1, \ldots ,{\text{ N}}} \right\}$ of hospitals, and for each hospital i, we have a corresponding error function $G_{i} \left( {\varvec{u}} \right)$, where ${\varvec{u}}$ is the model vector having dimensions $\delta ,{ }{\varvec{u}} \in R^{\delta }$. The optimization problem is constructed as follows^39^,

1 $$ \mathop {\min }\limits_{{ {\varvec{u}} \in R^{\delta } }} \frac{1}{H}\mathop \sum \limits_{i = 1}^{H} G_{i} \left( {\varvec{u}} \right) $$

where ‘H’ represents the number of hospitals. We want to find the u that minimizes the above equation. All of the hospitals have their own set of devices ${\mathcal{H}}_{D} \left( i \right)$ such that $i \in {\mathcal{H}}_{D}$ and $\left| {{\mathcal{H}}_{D} } \right|$ is the number of devices. Next, we define a new dataset $E_{i} { }$ as $E_{i} = \left\{ {{\varvec{x}},{\varvec{y}}} \right\}_{i} { }$ for the ith hospital where ${\varvec{x}}$ are the measurements and ${\varvec{y}}{ }$ are the labels. We now assume as follows.

All devices of the ith hospital can access the entire dataset Ei of that respective hospital.

We have to partition $E_{i}$ into $H_{D} \left( i \right)$ smaller subsets, $E_{i} = \left\{ {\left\{ {{\varvec{x}}_{1} ,{\varvec{y}}_{1} } \right\}, \ldots ,{ }\left\{ {{\varvec{x}}_{{{\mathbf{H}}_{{\mathbf{j}}} }} ,{ }{\varvec{y}}_{{{\mathbf{H}}_{{\mathbf{j}}} }} } \right\}} \right\}$ with the constraint that $\bigcap\limits_{k = 1}^{{{\text{H}}_{{\text{j}}} }} {e_{k} } = \emptyset { }$ such that $e_{k} \in E_{i}$, in simpler words, all partitions are disjoint there exists no common element between them. This is done to deal with the decentralized framework.

A model ${\varvec{u}}$ is received by the devices from the hospital and is updated using a Stochastic Gradient Descent (SGD) optimization algorithm, the learning rate & the number of epochs for every single device are the same.

The updated model is denoted as ${\varvec{u}}_{{\varvec{j}}}$ for the jth device. The error of the jth model for the kth subset of the dataset is defined as $l_{jk} \triangleq { }l\left( {{\varvec{u}}_{{\varvec{j}}} ;e_{k} } \right)$ where $e_{k} \in E_{i}$.

Next, we define the mean loss of our model over the entire dataset for the ith hospital as

2 $$ l_{j} = \frac{1}{{H_{D} \left( i \right)}}\mathop \sum \limits_{k = 1}^{{\left| {E_{j} } \right|}} l_{jk} $$

The loss of each subset is calculated and summed for the given model j.

Now we can define our original $G_{i} \left( {\varvec{u}} \right)$ as the sum of individual losses $l_{j}$. Then the loss for the ith hospital is

3 $$ G_{i} = \frac{1}{{H_{D} \left( i \right)}}\mathop \sum \limits_{j = 1}^{{H_{D} \left( i \right)}} \begin{array}{*{20}c} - \\ {l_{j} } \\ \end{array} = \mathop {\frac{1}{{H^{2}_{D} \left( i \right)}}}\limits^{{\mathop {\min }\limits_{{{\varvec{u}}_{1} , \ldots ,{\varvec{u}}_{{H_{D} \left( i \right)}} }} G_{i} }} \mathop \sum \limits_{j = 1}^{{H_{D} \left( i \right)}} \mathop \sum \limits_{k = 1}^{{\left| {E_{j} } \right|}} l_{jk} $$

For the ith hospital, the loss is the average of all the losses for $H_{D}$ devices in the hospital. $l_{j}$ as mentioned before is the loss of the jth model for the ith hospital.

Now we write our problem in terms of minimizing all the loss functions $G_{i}$ individually, where we find the model ${\varvec{u}}_{{\varvec{j}}}$ which minimizes the loss function. We now focus on the subsets instead of minimizing the sum of losses, this problem is handled as the primal assignment problem.

At the end of the training and testing process, the generalized adaptive global model of the proposed intelligent system is uploaded to the cloud for validation purposes. In the last phase of the proposed model, a validation layer is presented for the validation of new patients’ data on the trained adaptive model. In this layer, data is received from smart devices on run time, sent to a raw database, and preprocessed. After preprocessing, preprocessed image data is sent to a trained adaptive model for the classification of multidisciplinary cancer disease. If a multidisciplinary cancer disease exists, our proposed intelligent system will prompt with the label of a class of cancer disease in the patient’s body and recommend the patient to consult with a specialized doctor, else it will discarded.

A bipartite graph can be used to represent the relationship between hospitals and medical devices, where one set of vertices represents the hospitals and the other set represents the medical devices. An edge between a hospital vertex and a device vertex would indicate that the hospital has acquired or is using that device. The specific purpose of using a bipartite graph in this context is to analyze and optimize the allocation of medical devices to hospitals.

**Figure 2:**
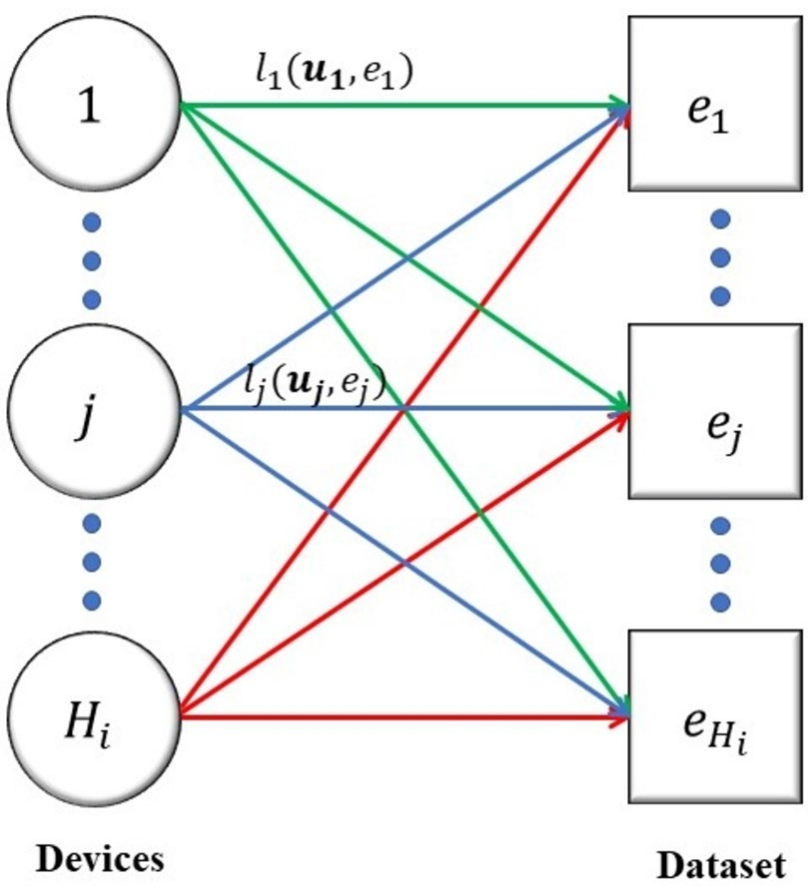
Dataset allocation of smart hospital i.

By representing the hospitals and devices as separate sets of vertices and using edges to represent their relationships as shown in Fig. 2, we can easily identify which devices are being used by which hospitals, and which hospitals have unmet device needs.

By using a bipartite graph, we can distribute the whole dataset into subsets as per the number of devices across all hospitals. This can help healthcare systems optimize their device allocation strategies to ensure that devices are being used efficiently and effectively.

Consider a bipartite graph ${\mathcal{G}}_{i}^{a} = \left\{ {H_{D} \left( i \right),{ }E_{i} ;Q_{i}^{a} } \right\}$ where $Q_{i}^{a}$ is the complete set of edges and the edge $\left( {j,k} \right) \in Q_{i}^{a}$ occurs if & only if, jth smart device can be allocated to the kth subset as shown in Fig. 2. We introduce a binary decision variable $d_{jk} \in \left\{ {0,1} \right\}$ for each edge $\left( {j,k} \right)$, which associates the kth subset to the jth device. The device is assigned to the subset of the variable that takes on the value of 1 and it is not assigned if it takes on the value of 0.

Now we model the problem as a multi-assignment problem

$$ \mathop {\min }\limits_{{{\varvec{d}} \ge 0}} \frac{1}{{H^{2}_{D} \left( i \right)}}\mathop \sum \limits_{{\left( {j,k} \right) \in Q_{i}^{a} }} l_{jk} d_{jk} $$

Subject to

4 $$ \mathop \sum \limits_{{\{ k|\left( {j,k} \right) \in Q_{i}^{a} \} }} d_{jk} , \forall j \in \left\{ {1, \ldots , H_{i} } \right\} $$

$$ \mathop \sum \limits_{{\{ j|\left( {j,k} \right) \in Q_{i}^{a} \} }} d_{jk} , \forall k \in \left\{ {1, \ldots , H_{i} } \right\} $$

where ${\varvec{d}} = \left[ {d_{11} ,d_{12} , \ldots ,{ }d_{{H_{D} \left( i \right),{ }H_{D} \left( i \right)}} } \right]^{T} { }$ is the vector of resulting variables. Similarly, the limitations illustrated that every device is to be allocated to a subset & this subset is to be allocated to a device.

**Figure:**
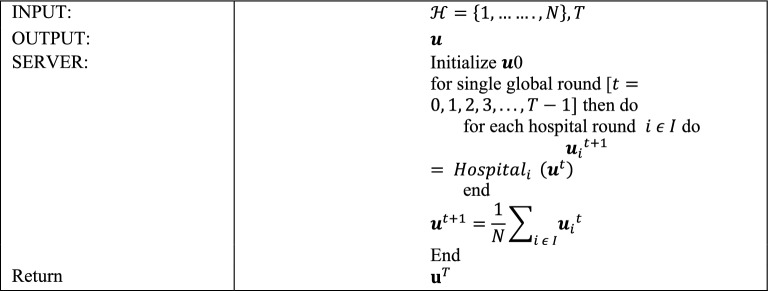
**Algorithm 1**: Centralized server.

**Figure:**
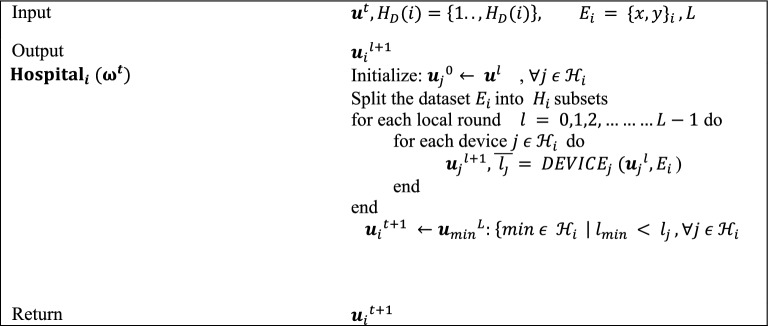
**Algorithm 2**: Smart hospitals.

Assume $G_{i}^{a}$ is complete. Also, note that $H_{D}^{2} \left( i \right) = \left| {Q_{i}^{a} } \right|$ and $d_{i} = H_{D} \left( i \right)$.

The issue is written in matrix form, succeeding the normal linear programming arrangement:

5 $$ \mathop {\min }\limits_{{{\varvec{d}} \ge 0}} {\mathbf{\mathcal{L}}}^{T} {\varvec{d}} $$

Focus to $${ }A{\varvec{d}} = {\varvec{b}}$$.

Where ${\mathcal{L}} \in {\mathbb{R}}^{{H_{D}^{2} \left( i \right)}}$ denotes the losses vector ${\mathcal{L}} = \left[ {l_{11} ,l_{12} , \ldots ,{ }l_{{H_{D} \left( i \right)H_{D} \left( i \right)}} } \right]^{T}$, and ${\varvec{d}} \in {\mathbb{R}}^{{H_{D}^{2} \left( i \right)}}$ the vector of decision variables. $A \in {\mathbb{R}}^{{d_{i} { } \times H_{D}^{2} \left( i \right)}}$ are the limitations of matrix and ${\varvec{b}} = {\mathbb{R}}^{{d_{i} }} { }$ is a vector of ones.

The network of $H_{D} \left( i \right)$ devices of the hospital i are modeled by a directed graph (digraph) ${\mathcal{G}}_{i}^{c} = \left( {{\mathcal{H}}_{i} ,Q_{i}^{c} } \right).$ ${\mathcal{H}}_{i}$ is the set of devices and $Q_{i}^{c}$ is the set of edges such that it is a subset of the Cartesian product of $H_{D} \left( i \right)$, $Q_{i}^{c} \subseteq H_{D} \left( i \right) \times H_{D} \left( i \right)$, where $\left( {j,k} \right) \in Q_{i}^{a}$ if the nearby edge moves from j to devise k.

In the first Algorithm, the vector W_0_ of the model is arbitrarily prepared and delivered to each hospital, beginning with the centralized server. The numeral of the universal aggregation round is denoted by T. When the model arrives at the hospital, the local training phase begins, as indicated in the smart health Algorithm. When the local training of the model is finished, the hospital chooses the local model with minimum loss and transfers it to a centralized server for universal aggregation. During this step, every single model is evaluated, and only the one that has the best performance on the entire dataset is forwarded to the centralized server.

Each smart hospital organizes the local training operation in Algorithm 2 by adjusting the accessible hospitals’ devices and it also splits the image dataset. The numeral of local training cycles is L, the number of devices is H_D (i), and the hospital dataset is Ei. During universal round t, hospital i obtains the universal Wt model from the central server and uses it to initialize the local model wj0 of each device j. The hospital distributes the model to each device before the first local round and allows permission to the complete segregated dataset Ei.

Following that, the hospital collects the updated local model wjl+1 as well as the related average loss j. Lastly, following the last local round of each hospital, each hospital returns the model having the lowest loss to the server.

In the third Algorithm, each device generates its loss vector Lj, the entries of which correspond to the estimated losses of model wlj on each subset. The initialization of the Decentralized Optimization function is done with Lj and Ei. In federated learning, decentralized optimization entails jointly training a global model on several decentralized devices without sharing raw data. First, a global model is transmitted to the participating devices after being set up on a central server. After that, each device trains the model independently using its local data, calculating gradients that indicate parameter changes for better results. To maintain privacy, only these gradients are sent back to the central server as opposed to raw data. These gradients are combined by the central server, which then modifies the global model. Until convergence, this repetitive sequence of global model update, communication, aggregation, gradient computation, and local training is repeated. By utilizing a decentralized method, devices can overcome privacy concerns in collaborative machine learning by improving models while maintaining localized data. The function handles the multi-assignment problem by executing the Distributed Simplex algorithm^40^. α is a scalar that reflects the assignment problem solution; in other words, it determines which subset of the dataset will be trained in the upcoming local cycle.

**Figure:**
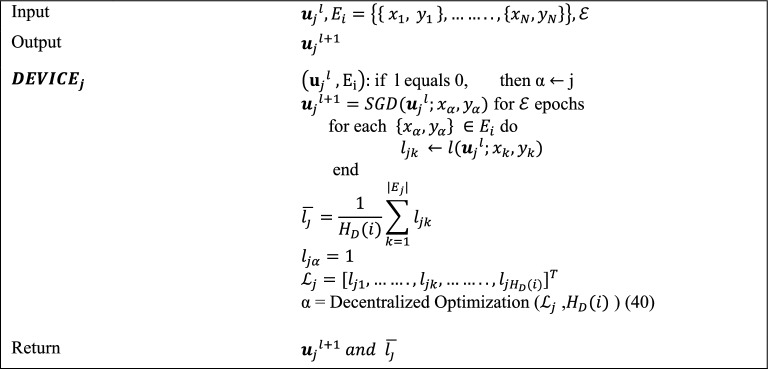
**Algorithm 3**: Smart hospital device.

The initial local cycle is started with the value j, which corresponds to the device numeral, such that all the devices train their models at subset j of the dataset. In this technique, an updated model ${\varvec{w}}_{j}^{l + 1} = {\varvec{w}}_{j}^{l} - \eta \nabla l\left( {w_{j}^{l} ;x_{\alpha } ,y_{\alpha } } \right)$ is computed on the subset chosen by using the Stochastic Gradient Descent^41^. E denotes the numeral of epochs, and $\eta$ represents the learning rate of the model. We reprimand the conclusion to train the model in the earlier selected subset before the next local round by assigning the extreme value ‘1’ to the loss j, which resembles the preceding choice.

The application of Convolutional Neural Networks (CNNs) is essential for improving the precision and effectiveness of cancer categorization. In particular, CNNs are essential for the interpretation of complex medical images from a variety of sources, including histology slides, CT scans, and MRIs. They are skilled at recognizing patterns suggestive of various cancer kinds because of their capacity to automatically extract hierarchical features. CNNs can be installed throughout dispersed healthcare facilities in the framework of Adaptive Federated Learning, facilitating cooperative model training without requiring the exchange of private patient information. This decentralized method complies with healthcare confidentiality regulations. The integration of CNNs into this multidisciplinary framework for classifying cancer diseases within Healthcare Industry 5.0 demonstrates the dedication to utilizing cutting-edge technology for accurate and customized cancer diagnosis, ultimately leading to better patient outcomes.

**Figure 3:**
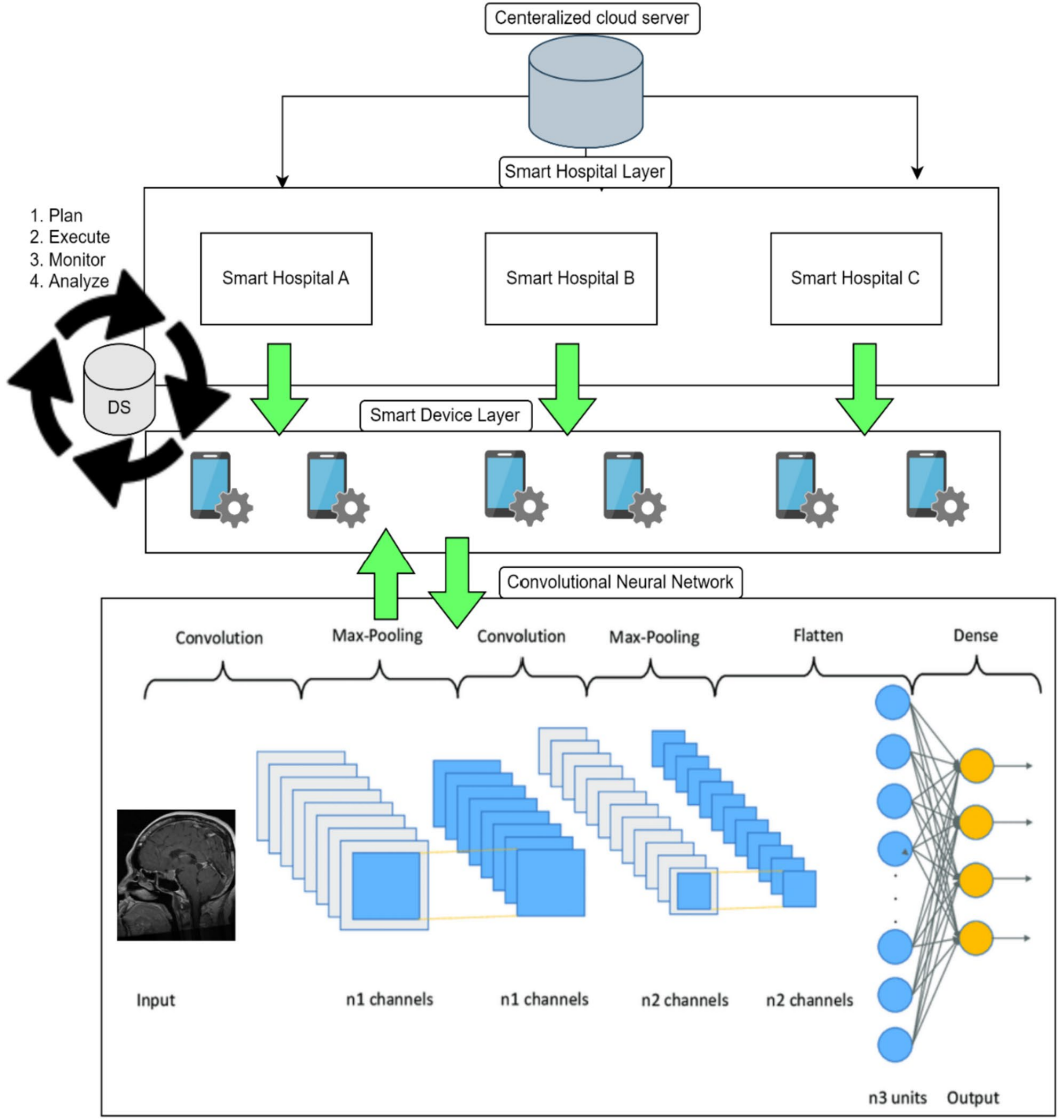
Graphical representation of flow chart hierarchy of proposed model.

A thorough case study can shed light on how this strategy is used in real-world healthcare situations and how beneficial it is. The graphical representation of the proposed model hierarchy between different layers is shown in Fig. 3. Local optimization is carried out in the layer of smart devices, and the suggested model is trained and displayed in Fig. 3 of the Convolutional Neural Network (CNN). The goal of the communication between all of these levels is to create a generalized adaptive federated learning model. To create a new master model, the weights of all the local models are shared with the global model. This approach continues until the global model and the learning criteria are the same. Finally, for testing and validation, we provided the cloud server with the generalized global model. The main advantage of using CNNs in federated learning is their ability to learn complex representations from raw input data, making them well-suited for tasks such as image recognition. Additionally, CNNs are relatively lightweight and can be trained efficiently on mobile devices, making them well-suited for federated learning scenarios. To support the suggested model's functionality, we used reputable datasets for the multidisciplinary classification of cancer diseases in this study as a case study. Three datasets are combined to provide the simulation and findings.

### Dataset

We used multiple cancer datasets^38^, containing eight (08) types of cancer image datasets. We considered only three cancer diseases in our case study; (1) brain cancer, (2) breast cancer, and (3) kidney cancer. Brain cancer is divided into three sub-folders of different types of brain cancer. All these subfolders contain a total of 15,000 images and 5000 images in each class of cancer. Breast cancer and kidney cancer have two subclasses. Both these datasets are comprised of 20,000 images and each dataset has a 10,000 images dataset. The data fusion approach is used for making an enriched dataset for the classification of deadly multidisciplinary cancer diseases. This fused dataset contained 35,000 images of seven (07) subtypes of cancers. The experimentation and classification of cancer are done through an adaptive federated learning approach empowered with CNN as discuss above.

## Results and discussion

We examine our algorithm's performance by running simulation tests with “i.i.d” data. The setup we demonstrated in MATLAB 2020a with the deep machine learning (DML) toolbox comprises three (03) hospitals and six (06) devices. We select a convolutional neural network model and modified multiple cancer datasets used for training and validation, which include 35,000 synthetic grayscale images of the brain, breast, and kidney, along with seven (07) subclasses. The total dataset is divided into 70% and 30% for training and validation purposes, respectively. The CNN contains a 28 × 28 × 1 input layer, three convolution 2D layers with Relu and Max pooling layers between them, and at the end for classification purposes, a softmax function is used in the fully connected layer.

First, we partition the dataset into hospitals of equal size, and then further into smart devices in each hospital. Each hospital has a unique subset, but all have the same number of photos and metadata. We start each hospital and gadget with the same CNN model. We made the comparison of our proposed method to federated averaging (FA) concerning an equal sum of devices and the iterations, i.e. the numeral loop involved for FA is the same as total global aggregation * local training rounds (T*L). The examination involves averaging the resultant loss of every smart device (l_j) across all hospitals.

The implementation environment and training parameters are shown in Tables 3 and 4 respectively.

**Table 3:** Implementation environment.

Tool/device Name	Description
Desktop System	Windows 10 pro N (Version 21H1)
Processor	Intel(R) Core (TM) i7-4770 CPU @ 3.40GHz 3.40 GHz
RAM	18.0 GB
MATLAB	2020a

**Table 4:** Training options and parameters.

Training preferences	Considerations
Size of image	28*28*1
Number of hospitals	03
Number of devices	06
Number of epochs	10
Iterations per epoch per device	10*10
Total iterations	10*10*10
Initial learning rate	0.001
Momentum	0.9
Solver	SGDM
Execution Environment	Auto
Mini batch size	128
Shuffle	every-epoch
Validation frequency	1

As shown above in Fig. 1 of our proposed model, the proposed adaptive FL model predicts outcomes. It is indicated by the value according to the class name and subclass name that a cancer disease has been identified. Table 1 displays the merged dataset for the three cancer types. The dataset has a total of seven (07) sub-diseases of cancers. In experimentation and simulation, we considered the fused dataset for the classification of multidisciplinary cancer disease. We have divided the whole dataset into 70% for training purposes, and 30% for validation purposes.

**Figure 4:**
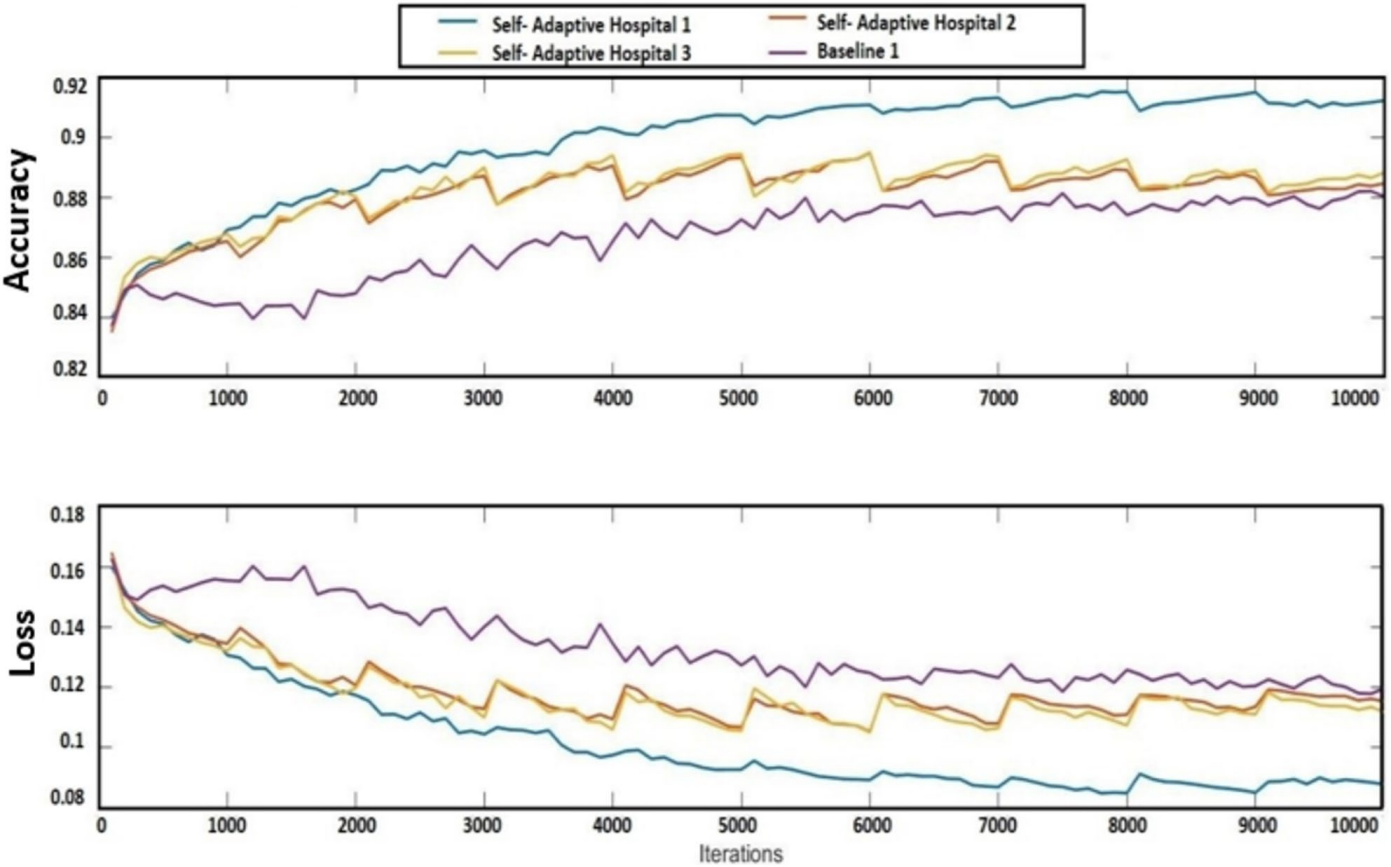
Proposed AFL model performance with each hospital’s vs conventional FL model.

In Fig. 4, the proposed FL model performance is compared with the conventional FL model graphically. In this paper, we have considered three (03) hospitals having 2 devices in each hospital. The accuracy of the proposed AFL model concerning each hospital is compared with the baseline model. The comparison is made with accuracy vs iterations. At the initial stage, the accuracy of both models was the same, but gradually the accuracy of the proposed model increased. As the number of iterations increased, so did the accuracy of all the hospitals. When compared to the other hospitals in the proposed AFL model, hospital 1 had the highest accuracy. Hospitals 1 and 2’s accuracy roughly reaches the same level, but it is still far greater than the base model.

**Figure 5:**
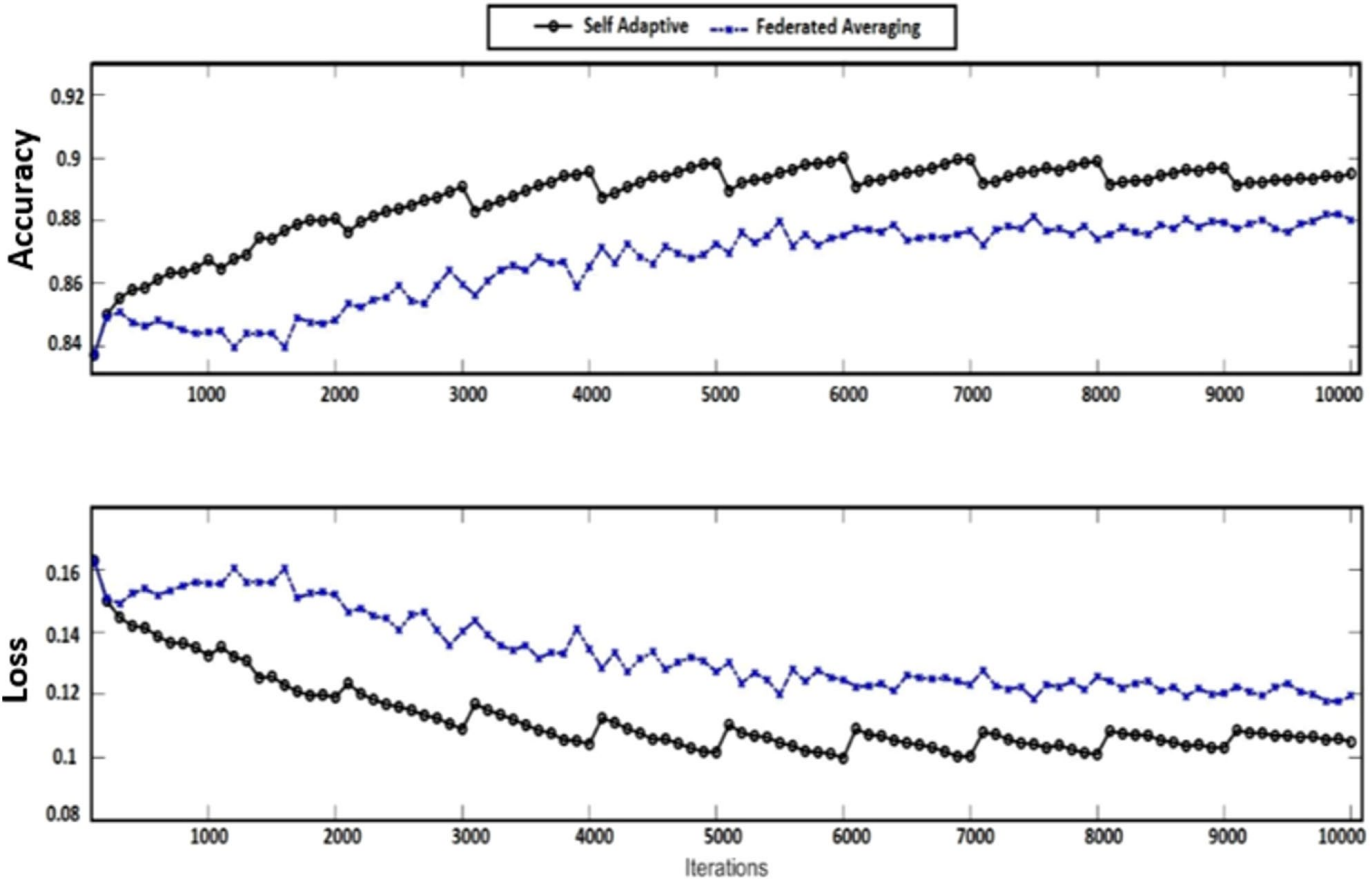
Proposed AFL model performance vs conventional FL model.

In the same way, the accuracy of the generalized proposed AFL model vs the conventional FL model is shown in Fig. 5. The comparison of losses of both models is also shown graphically in Figs. 4, and 5 which indicates the performance of each model. The confusion matrix of the proposed adaptive FL and FL model’s performance of the fused datasets at the training level are shown in Tables 5 and 6 with normalized form and similarly, Tables 7 and 8 respectively.

**Table 5:** Confusion matrix of AFL model at fused cancer dataset in the training phase.

	Class ID	Predicted class						
		1	2	3	4	5	6	7
True class	1	3482	0	18	0	0	0	0
	2	18	3464	18	0	0	0	0
	3	18	0	3482	0	0	0	0
	4	0	0	0	2616	884	0	0
	5	0	0	0	0	3500	0	0
	6	0	0	0	0	0	3500	0
	7	0	0	0	0	0	0	3500

**Table 6:**
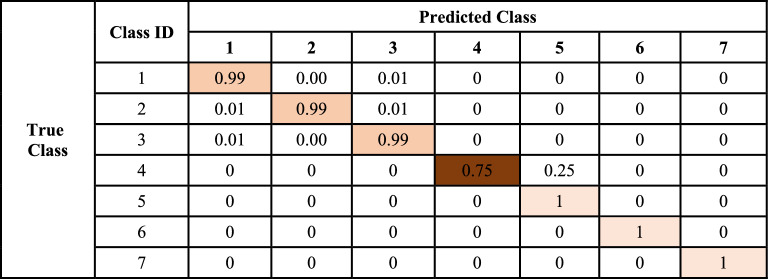
Normalized confusion matrix of AFL model at fused cancer dataset in the training phase.

**Table 7:** Confusion matrix of the FL model in the training phase.

	Class ID	Predicted class						
		1	2	3	4	5	6	7
True class	1	3085	343	72	0	0	0	0
	2	379	2941	180	0	0	0	0
	3	108	144	3247	0	0	0	0
	4	0	0	0	3031	469	0	0
	5	0	0	0	397	3103	0	0
	6	0	0	0	0	0	3482	18
	7	0	0	0	0	0	0	3500

**Table 8:**
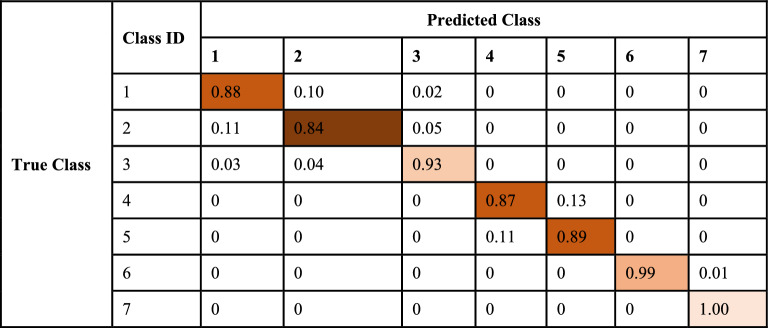
Normalized confusion matrix of FL model in the training phase.

According to subclass 5, 3500 photos are genuinely positive for the subclass 5 cancer disease, which is being actively monitored and indicates that problems with type “kidney tumor” have been noted. Not a single report is mislabeled as belonging to a different category of cancer. It is found that 3500 photos for subclass 6 of the cancer disease are genuinely positive. This subclass is closely monitored and indicates that the cancer type “breast benign” has been observed. There are no entries that are mislabeled as belonging to other cancer disease classes. According to subclass 7, 3500 photos are genuinely positive for the cancer disease subclass 7, which is being attentively monitored and indicates that type "breast malignant" problems have been noted. Not a single report is mislabeled as belonging to a different category of cancer.

During the training phase, the types of interdisciplinary cancer diseases were predicted using the adaptive FL model. The experiment uses 24,500 photos that are separated into seven categories with identically sized subclass names. According to the confusion matrix displayed in Table 5, 3482 photos are genuinely positive for Type 1 interdisciplinary cancer disease. This finding is consistent with the type of “brain glioma” difficulties that were noted. Just 18 data are mislabeled as belonging to different cancer illness classifications, indicating problems with brain tumors. 3464 photos are found to be genuinely positive for subclass 2 of cancer disease, which is being actively monitored and indicates that type “Brain menin” problems have been noted. Just 18 and 18 records, indicating brain forms of brain glioma and brain tumor concerns, respectively, are mis-projected as other classes of cancer condition. 3482 photos are found to be genuinely positive for subclass 3 of cancer disease, which is being actively monitored and indicates that problems with the cancer type “brain tumor” have been noted. Just 18 data are mislabeled as belonging to different cancer disease groups, indicating problems with brain gliomas of the cancer type. 2616 photos are found to be genuinely positive for subclass 4 cancer disease. This observation is closely monitored and indicates that the cancer subtype “kidney normal” has been identified. Merely 884 records are misclassified as a different type of malignancy, indicating problems with kidney tumors exclusively. The FL model prediction for the various interdisciplinary cancer conditions throughout the training period is shown in Tables 7 and 8. During the training phase, the 24,500 photos are split up into seven categories, each with a subclass name of the same length. According to Table 7 confusion matrix, which is strictly followed and indicates that type “brain glioma” concerns have been noticed, 3085 photos are genuinely positive for Type 1 of interdisciplinary cancer sickness. Just 343 and 72 records, respectively, are mislabeled as having problems with brain tumors and other kinds of cancer disease signaling brain menin. According to subclass 2, 2941 photos are genuinely positive for the cancer disease subclass 2, which is being attentively monitored and indicates that type “Brain menin” problems have been noted. Merely 379 and 180 records, indicating brain forms of brain glioma and brain tumor concerns, respectively, are misclassified as other classifications of cancer condition. 3247 photos are found to be genuinely positive for subclass 3 of cancer disease, which is being actively monitored and indicates that problems with the cancer type “brain tumor” have been noted. Just 108 and 144 data, which indicate brain glioma and brain menin problems, respectively, are mis projected as belonging to other kinds of cancer disorders. According to subclass 4, 3031 photos are genuinely positive for the cancer subtype “kidney normal, ” which is closely monitored and indicates that the subclass 4 cancer sickness has been identified. Merely 469 records are misclassified as a different type of malignancy, indicating problems with kidney tumors exclusively. It is found that, for subclass 5, 3103 photos are positive for subclass 5 cancer disease. These images are being monitored carefully and the type of “kidney tumor” problems have been noted. Just 397 records—signaling merely typical renal issues—are mistakenly projected as belonging to a different category of cancer sickness. 3482 photos are found to be genuinely positive for subclass 6 of cancer disease, which is continuously monitored and indicates that cancer type “breast benign” has been detected. Just 18 records are misclassified as belonging to other cancer types, indicating a malignant problem exclusive to the breast. According to subclass 7, 3500 photos are genuinely positive for the cancer disease subclass 7, which is being attentively monitored and indicates that type “breast malignant” problems have been noted. Not a single report is mislabeled as belonging to a different category of cancer.

In the validation phase, Table 9 presents the suggested adaptive FL model prediction for the various types of interdisciplinary cancer disorders and Table 10 represents its normalized form. The validation process makes use of the 1500 photos, which are split up into seven categories with identically sized subclass names. 1237 photos are genuinely positive for Type 1 interdisciplinary cancer disease, according to the confusion matrix displayed in Table 9, which is closely followed and indicates that type “brain glioma” difficulties have been noted. Merely 198 and 48 records are mislabeled as brain tumors, signaling brain menin, and other types of cancer sickness, respectively. According to subclass 2, 1017 photos are genuinely positive for the cancer illness subclass, which is being actively monitored and indicates that type “Brain menin” problems have been noted. The only records that are mislabeled as belonging to other kinds of cancer sickness are 229, 225, 1, 5, and 23. These records indicate brain gliomas, brain tumors, kidney tumors, benign breast concerns, and malignant breast issues, respectively. According to subclass 3, 1314 photos are genuinely positive for the cancer illness subclass, which is being actively monitored and indicates that problems with the cancer type “brain tumor” have been noted. Only 68 and 116 data, which indicate brain glioma and brain menin problems, respectively, are misprojected as belonging to other types of cancer disorders. Regarding subclass 4, it is noted that 1046 images are genuinely positive for subclass 4 cancer disease. These images are closely monitored and demonstrate the presence of the cancer subtype “kidney normal.”

**Table 9:** Confusion matrix of the proposed adaptive FL model in the validation phase.

	Class ID		Predicted class					
		1	2	3	4	5	6	7
True class	1	1237	198	48	0	0	0	17
	2	229	1017	225	0	1	5	23
	3	68	116	1314	0	0	0	2
	4	0	0	0	1046	451	0	3
	5	0	0	1	286	1212	0	1
	6	0	0	0	1	0	1475	24
	7	5	10	0	0	1	40	1444

**Table 10:**
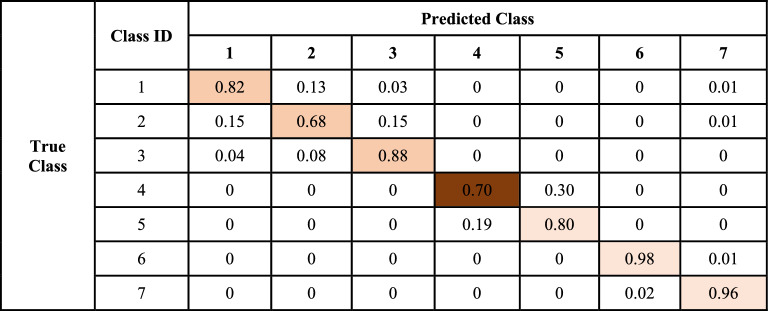
Normalized confusion matrix of AFL model at fused cancer dataset in the validation phase.

Merely 451 data are misclassified as a different type of malignancy, indicating problems with kidney tumors exclusively. It is found that, for subclass 5, 1212 photos are genuinely positive for subclass 5 cancer disease. These images are being monitored carefully and indicate that problems with type “kidney tumor” have been reported. Just 1, 286 and 1 records—signaling brain tumor, renal normal, and breast malignant issues—are misprojected as belonging to other subclasses of cancer conditions. 1475 photos are found to be genuinely positive for subclass 6 of cancer disease, which is closely monitored and indicates that the cancer type “breast benign” has been detected. Only records 1 and 24 are misclassified as different cancer disease subclasses, indicating a benign kidney and a malignant breast issue exclusively. It is found that, for subclass 7, 1444 photos are genuinely positive for the cancer disease subclass 7, which is being actively monitored and indicates that type “breast malignant” problems have been noted. Only records 5, 10, 1, and 40 are mislabeled as belonging to different subclasses of cancer disorders, indicating a malignant breast issue, a brain tumor, and meningitis in the brain.

The following metrics yield different statistical measurements for performance and comparison: F1-score, Accuracy, Precision, Misc. Rate, and Recall. These parameters are computed using the formulas in Eqs. (6)–(10)^42^ as indicated below.

6 $$ Accuracy \left( {Acc} \right) = \frac{TP + TN}{{ TP + TN + FP + FN }} $$

7 $$ Misclassification\; rate = \frac{FP + FN}{{ TP + TN + FP + FN }} $$

8 $$ Precision \left( {Pre} \right) = \frac{TP }{{ TP + FP }} $$

9 $$ Sensitivity\left( {recall} \right) = \frac{TP }{{ TP + FN }} $$

10 $$ F 1 Score \; = 2*\left( {\frac{Precision*Recall}{{Precision + Recall}}} \right) $$

Tables 11 and 12 present the training phase accuracy comparison between the proposed adaptive FL model and the standard FL model. As indicated in Tables 11 and 12, the comparison of these models also goes into detail on the precise, recall, F2 score, and individual obtaining accuracies of each class. Table 13 displays the extraction of the same comparison from an experiment conducted during the validation phase of the suggested adaptive FL model. Tables 11, 12, and 13 present an analysis of the preliminary results, which include accuracy, precision, recall, specificity, and F1 score.

**Table 11:** Overall performance of the conventional FL model in the training phase.

Class	Accuracy	Misc. Rate	Precision	Recall	F1 score
1	98.28%	1.72	0.99	0.98	0.99
2	98%	2.00	0.84	0.86	0.85
3	99.03%	0.07	0.93	0.93	0.93
4	99.10%	0.90	0.87	1	0.93
5	93.17%	6.83	0.11	0.46	0.18
6	94.03%	5.97	0.99	0.53	0.69
7	99.97%	0.03	1	0.99	1

**Table 12:** Overall performance of adaptive FL model in training phase.

Class	Accuracy	Misc. rate	Precision	Recall	F1 score
1	99.74%	0.26	0.99	0.99	0.99
2	99.83%	0.17	0.99	1	0.99
3	99.74%	0.26	0.99	0.99	0.99
4	95.79%	4.21	0.75	1	0.86
5	95.79%	4.21	1	0.8	0.89
6	100%	0	1	1	1
7	100%	0	1	1	1

**Table 13:** Overall performance of the proposed adaptive FL model at validation.

Class	Accuracy	Misc. rate	Precision	Recall	F1 score
1	94.62%	5.48	0.82	0.8	0.81
2	92.31%	7.79	0.68	0.76	0.72
3	95.62%	0.26	0.88	0.83	0.85
4	92.94%	7.16	0.7	0.78	0.74
5	92.94%	7.16	0.81	0.73	0.77
6	99.33%	0.67	0.98	0.97	0.98
7	98.80%	1.20	0.96	0.95	0.96

Table 14 presents an overall comparison of these models’ accuracies, confirming and validating the more highly-proposed FL model. In the provided datasets, the outcomes of our suggested adaptive FL model performed better. The proposed adaptive FL model’s overall accuracy has decreased marginally as a result of the many cancer conditions being combined. However, in the fused dataset of interdisciplinary cancer disease, the outcomes of our suggested approach outperformed the traditional FL model.

**Table 14:** Overall accuracy comparison of adaptive FL vs FL.

Proposed adaptive FL Model (Overall, Acc.)			FL model (Overall, Acc.)	
Dataset	Accuracy at training phase	Accuracy at validation phase	Accuracy at training phase	Accuracy at validation phase
Fused dataset	89.83%	83.29%	87.3%	80%

## Conclusions

It is difficult to predict human diseases, particularly cancer, to give better and more timely treatment. Cancer is a life-threatening condition that affects larger areas of the human body. We present a self-adaptive framework for federated learning of healthcare 5.0 automated systems in this study. Our method entails categorizing the healthcare 5.0 environment across three levels: central server, smart hospitals, and smart hospital devices. We model the architecture's self-adaptive behavior using error control loops. We characterize the training method as a multi-task issue and apply distributed optimization as a result. For the same number of communication rounds, the results of our technique indicate higher model accuracy than the typical federated averaging strategy. The proposed automated intelligent system for the healthcare industry 5.0 with adaptive FL is simulated using MATLAB 2020a. For multi-disciplinary cancer disease categorization in the smart healthcare industry 5.0, the suggested intelligent system with adaptive FL methodology achieved an overall 89.38%, which is greater than the standard FL model with 86%.

## Contribution and future work

The latest study has already presented several recommendation algorithms for the healthcare industry. This research makes a major contribution by providing an automated adaptive FL system for the finest Trans disciplinary cancer illness prediction in the healthcare industry 5.0. Despite these current discoveries, the proposed design might be further studied. For example, several latest deep CNN approaches and the varieties of datasets will be examined to make a more accurate and authenticated FL model. Furthermore, implementing the design in a real-world healthcare setting may present unexpected obstacles. As a result, one of our next initiatives is to integrate with current healthcare industry 5.0 solutions.

## Data Availability

The original contributions presented in the study are included in the article/supplementary material, further inquiries can be directed to the corresponding author/s.
